# Use of Biochar to Improve the Sustainable Crop Production of Cauliflower (*Brassica oleracea* L.)

**DOI:** 10.3390/plants11091182

**Published:** 2022-04-27

**Authors:** Daniela Losacco, Marina Tumolo, Pietro Cotugno, Natalia Leone, Carmine Massarelli, Stefano Convertini, Angelo Tursi, Vito Felice Uricchio, Valeria Ancona

**Affiliations:** 1 Water Research Institute-Italian National Research Council (IRSA-CNR), 70132 Bari, BA, Italy; marina.tumolo@ba.irsa.cnr.it (M.T.); natalia.leone@ba.irsa.cnr.it (N.L.); carmine.massarelli@ba.irsa.cnr.it (C.M.); vito.uricchio@ba.irsa.cnr.it (V.F.U.); 2 Department of Biology, University of Bari, 70126 Bari, BA, Italy; pietro.cotugno@uniba.it (P.C.); angelo.tursi@uniba.it (A.T.); 3 ReAgri S.r.L, 74016 Massafra, TA, Italy; sconvertini@reagri.it

**Keywords:** *Brassica oleracea* L. var. *botrytis*, nitrogen fertilization, soil properties, biochar, plant yield

## Abstract

In agriculture, biochar (B) application has been suggested as a green technology to reduce nitrate pollution from agricultural origins and improve crop yield. The agronomic impact of B use on soil has been extensively studied, while knowledge of its possible effects on horticultural cultivation is still scarce. A greenhouse experiment was conducted to evaluate the effect of using biochar in soils treated with two different rates of nitrogen fertilizers on soil properties and nitrogen (N) leachate. This study also investigated the vegetative parameters during the crop growing season of *Brassica oleracea* L. var. *botrytis*. Soil mesocosms were set up to test the following treatments: untreated/control (C); normal dose of N fertilizer (130 kg N ha^−1^) (ND); ND+B; high dose of N fertilizer (260 kg N ha^−1^) (HD); and HD+B. Principal component analysis and cluster analysis were exploited to assess biochar’s ability to reduce nitrate leaching and enhance soil–vegetative properties. Biochar addition affected the soil chemical properties of the fertilized microcosms (ND and HD). Biochar increased the $NH_{4}^{+}$ content in HD soil and the $NO_{3}^{-}$ content in ND soil by 26 mg/L and 48.76 mg/L, respectively. The results showed that biochar application increased the marketable cauliflower yield. In ND+B and HD+B, the curd weight was 880.68 kg and 1097.60 kg, respectively. In addition, a small number of nitrogenous compounds in the leachate were quantified in experimental lines with the biochar. Therefore, biochar use improves the marketable yield of horticulture, mitigating the negative impacts associated with the mass use of N fertilizers in agriculture.

## 1. Introduction

Cauliflower (*Brassica oleracea* L. var. *botrytis*) is one of the most important horticultural species belonging to the Brassicaceae family [1], grown on more than 15,670 ha in Italy alone [2]. Cauliflower is a horticultural species mainly cultivated in the Mediterranean area, with Italy as the main producer, with about 4 million tons being produced in 2019 [2]. Crop growth and yield are closely related to nitrogen (N) availability, and N uptake rate varies according to vegetative phase. To gain a high yield and good quality of curd, the cultivation demands a constant, generous nitrogen supply [3], with absorption rates in the order of 130–150 kg N ha^−1^ year^−1^ [4].

N is applied according to empirical standards, and the mass use of nitrogen fertilizer is directly associated with increases in agricultural yield, despite the pollution of underground crops due to leaching phenomena [5]. High N fertilization is a standard practice used by farmers for vegetable management to achieve a high yield of commercial product. The negative environmental effects of mineral-origin nitrogen are of global interest, and there is increasing determination to mitigate its use for agricultural purposes [6] and to investigate new sustainable agronomic strategies [7].

Nitrogen (N) is an important element to plants, and is necessary in a number of physiological processes including photosynthesis, metabolite biosynthesis, and flowering [8,9]. The effect of nitrogen fertilizer on crop growth depends on the quantity and availability of inorganic N concerning nitrogen crop demand. Nitrogen farming practices are associated with specific agronomic factors, such as the amount and percentage of total N applied, the decomposition percentage of organic fractions, and the carbon–nitrogen ratio of available fractions for decomposition [10]. The culture of nitrogen demand usually follows a sigmoidal model, and is defined as the absorption of N during a useful period for maximum dry matter production [11]. Nitrogen content, such as nitrate concentration, can change within species, cultivars, and even genotypes with different ploidy [12]. Furthermore, nitrate content changes in the different plant tissues, decreasing as follows: petiole > leaf > stem > root > inflorescence > tuber > bulb > fruit > seed [13,14]. Nitrate-accumulating horticulture species typically belong to the Brassicaceae family, with nitrogen absorption mainly localized in the leaves, with values of 69–74% in cauliflowers [15].

In recent years, an agricultural challenge has been to obtain sufficient quantities of plant foods while minimizing the environmental impact of sustainable agriculture [16]. Modern agricultural production requires sustainable fertilizer management practices through the application of appropriate rates and sustainable agricultural strategies. Agricultural mitigation measures comprise both traditional and innovative techniques, for the simultaneous protection of environmental matrices and to increase the efficient use of N compounds [17].

Biochar is an aromatic and carbon-rich solid by-product predominantly found from agricultural residues pyrolyzed in an environment with limited oxygen [18]. It can be used in farms, in manure treatment, as a composting additive, and possibly for soil amendment [19]. In modern agriculture, biochar is mainly applied to increase crop yields and to improve the soil’s physical, chemical, and biological properties. The utilization of biochar to agricultural soils contributes to element storage, but at the same time acts as a fertilizer [20], improving soil fertility and enhancing agricultural productivity. The effect of biochar amendment on soil nutrient substances positively affects nutrient retention, especially in soils with poor ion-retention capacities [21]. Biochar use improves the content of total C, organic C, total N, available P, Ca, Mg, Na, and K in soil [22]. Several studies have shown that biochar carries out an important role in the soil’s retention of N, reducing inorganic N-leaching [23,24,25], and thus increasing the bioavailability of N for plants.

Biochar enhances both plant growth and crop yield, increasing food production and sustainability in zones with limited natural resources, insufficient water, and restricted access to fertilizers [26]. The high porosity and surface/volume ratio of biochar improves nutrient absorption by the plant [22]. In addition, biochar use increases the ability to retain soil moisture and nutrients [27], increases the soil’s pH [28], and provides essential nutrients to plants to reduce the need to apply chemical fertilizers [29]. Biochar is a promising green fertilizer capable of mitigating nitrogen losses from the soil and changing the N dynamics in agricultural soils.

Several studies have examined the effects of biochar co-applied with chemical and organic nitrogen fertilizer [30,31,32,33,34]. Specifically, biochar interaction with mineral or organic nitrate has recently been shown as a fundamental mechanism for the promotion of plant growth, with the slow release of N from biochar [35,36].

The objective of the present study was to quantify the release of nitrogen compounds (nitrate, ammonium) from biochar-amended soil in comparison to untreated (Control—C) soil for cauliflower (*Brassica oleracea* L.) mesocosms. Moreover, the influence of the biochar fertilization strategies on the soil properties and the yield of the horticultural products were assessed.

## 2. Results

### 2.1. Effect of Biochar-Amended Soil with N Fertilizers on Soil Chemical Properties

Table 1 shows the changes in soil chemical properties at the end of the cauliflower growing season. In comparison to the control (no treatment) and soil without B, biochar-amended soil with 130 kg N ha^−1^ increased the C/N ratio and $NO_{3}^{-}$ concentration by 130.61 and 48.76 mg/L, respectively. However, electrical conductivity (EC), water content, available phosphorus (P), total organic carbon (TOC), total nitrogen (N), total carbon (C), and $NH_{4}^{+}$ amount decreased after treatment with biochar. In the biochar-amended soil treated with 260 kg N ha^−1^ of N fertilizers, TOC, total C, and $NH_{4}^{+}$ increased. Biochar application did not change the level of pH, available P, or total N contents, while EC, water content percentage, and $NO_{3}^{-}$ amount decreased in soil amended with the green compound. The soil pH values were acidic, as the soil type used in the experiment was peat (pH = 4.97–5.14) (Table 1). Under the experimental conditions of this study, treatments with or without biochar did not modify the soil’s pH. The EC values of biochar-amended soils with conventional and high fertilizing doses of N were 2.66 dS/m^−1^ and 3.57 dS/m^−1^, respectively. The highest EC was found in HD with and without biochar (4.46 and 3.57 dS/m^−1^). In contrast, the lowest EC was ascertained in untreated (C) soil and in soil fertilized with 130 kg N ha^−1^. The application of biochar in the fertilized conventional dose soil increased the EC from 2.32 dS/m^−1^ (ND) to 2.66 dS/m^−1^ (ND+B). The highest available P was found in ND experimental condition. No significant differences in P content (20 mg kg^−1^) in the mesocosms treated with 260 kg N ha^−1^, with or without biochar, were observed. The lowest content of available P was detected in biochar-amended soil with 130 kg N ha^−1^, probably due to the increased P uptake by the cauliflower. Compared to the control, the level of TOC in all treatments increased. The highest value was determined in ND (21.89%), followed by HD+B (16.74%), and ND+B (14.83%). The total N and C contents of treated soil ranged from 0.2 to 0.4% (total N), and from 25.5 to 34.99% (total C). A high total N content of 0.4% was found in soil fertilized with 130 kg N ha^−1^, while the total C content was high in ND (34.99%) and HD+B (30.75%). The slightest level of total C content was quantified in ND+B (25.5%). The C/N ratio of biochar influenced the activity of the microorganisms and the total N in the soil. The C/N ratio of treated soil changed from 124.71 (ND) to 130.61 (ND+B). The application of biochar in both nitrogenous fertilizing assays increased the C/N ratio.

The concentration of $NH_{4}^{+}$ in the different treatments considerably changed with the type of N fertilizer and amount. The content $NH_{4}^{+}$ in soil, during the cauliflower growing season, ranged from 7.0 to 4.62 mg/L in the soil fertilized with 130 kg N ha^−1^ (ND) and ND+B, respectively; soil $NH_{4}^{+}$ content ranged from 18.22 to 26 mg/L in the soil fertilized with 260 kg N ha^−1^ (HD) and HD+B treatments, respectively. The concentrations of soil $NO_{3}^{-}$ also varied, ranging from 22.01 mg/L in ND to 48.76 mg/L in ND+B across the whole experiment. The $NO_{3}^{-}$ concentrations ranged from 99.69 mg/L to 77.63 mg/L in the high N fertilizer treatments.

#### Principal Component Analysis and Cluster Analysis of Soil

Principal component analysis (PCA) was applied to identify the relationships between the investigated soil chemical properties and the soil samples for the different treatments. PCA scores were then submitted to cluster analysis (CA) to group the individual samples. Two principal components (PC) were specified, explaining about 57% (PC1: 38.2%; PC2: 19.21%) of the total soil data variance (Table 2).

**Figure 1:**
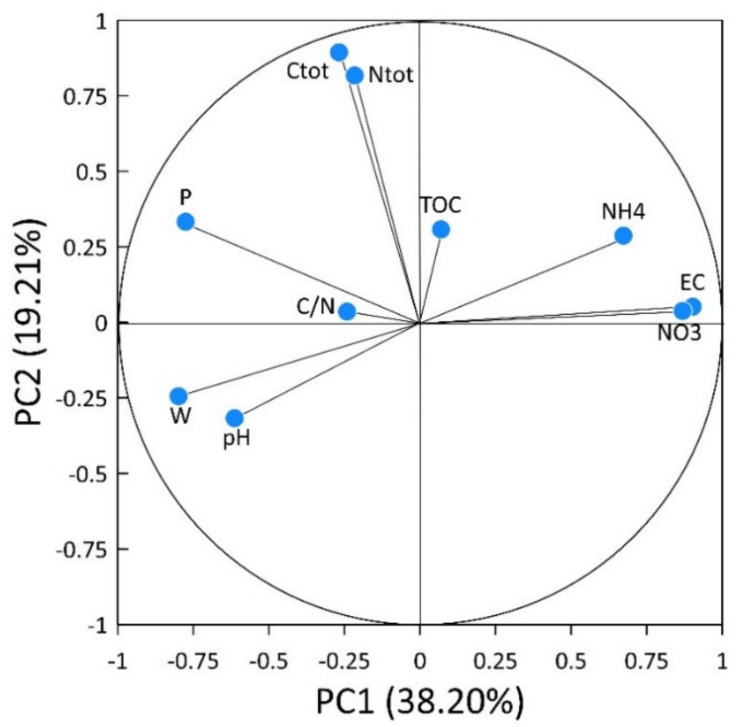
Principal component plot for the chemical soil properties at the end of cauliflower growing.

The distribution of variables in the plane, defined by the two components (Figure 1), shows that many of the variables contributed strongly to PC1. The parameters of electrical conductivity (EC) (0.902), $NH_{4}^{+}$ concentration (0.673), and $NO_{3}^{-}$ quantity (0.869) were positively correlated to PC1, while pH (0.614), water content percentage (0.799) and available P (0.775) were negatively correlated to PC1 (Table 2). Only total N (0.817) and total C (0.894) were positively correlated to PC2 (Table 2). Other major factors explained small fractions of the data variance, and were characterized by an absence of meaning.

**Figure 2:**
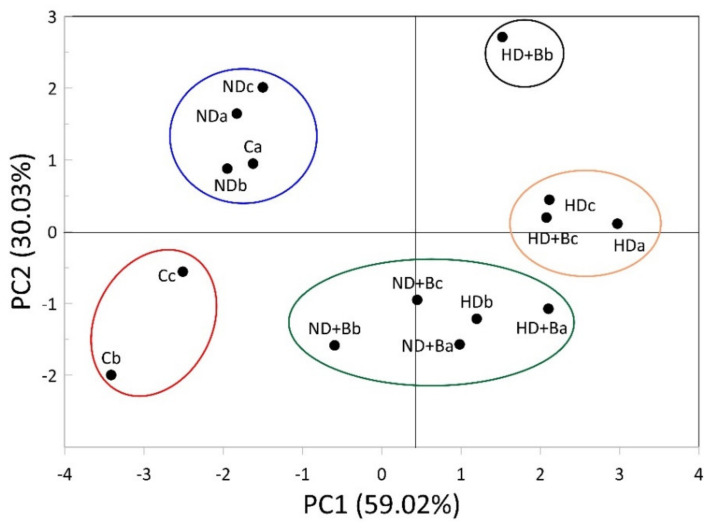
Hierarchical cluster analysis for the soil properties of different N fertilizer treatments with and without biochar. C—control; ND—normal dose; ND+B—normal dose with biochar; HD—high dose; HD+B—high dose with biochar; a-b-c indicate the triplicate samples.

The cluster analysis (CA), applied to the scores of principal component analysis (PCA), allowed the identification of five clusters (Figure 2). The first cluster (C1) included one replicate control soil (Ca) and the soils treated with 130 kg N fertilizer (NDa, NDb, and NDc). Moreover, Figure 2 shows the second and third clusters including C_b and C_c control soils, and the soils treated with 130 and 260 kg N fertilizer with biochar (ND+Ba, ND+Bb, ND+Bc, HDb, and HD+Ba), respectively. The fourth cluster included two soils treated with double-dose fertilizer (HDa, HDc) and one with double-dose fertilizer with biochar (HD+Bc). Finally, the fifth cluster comprised only one soil treated with double-dose N fertilizer with biochar (HD+Bb).

The soil chemical properties related to different biochar-amended soil treatments showed high significance (*p* = 0.000701 ***). Because the multivariate test found significance, the ANOVA was performed to determine which of the variables and factors influenced the significance (Table 1).

### 2.2. Ammonium and Nitrate Assessment in Water-Leached Samples

**Figure 3:**
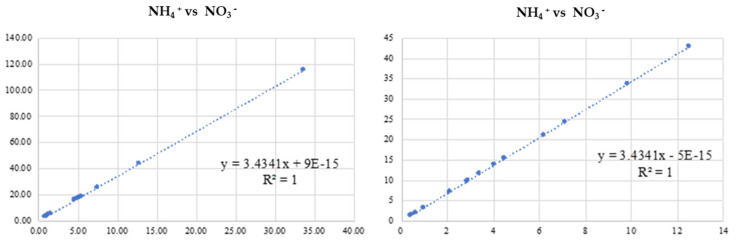
Relationship between nitrogen compounds (mg/L) in water-leached samples after the first N fertilizing practice (**on the left**), and the second N fertilizing practice (**on the right**).

In the present study, a simple linear regression analysis was carried out to explore the relationships between the nitrogenous compounds after two N fertilizer treatments (Time 1 and Time 2) in the water-leached samples (Figure 3). $NH_{4}^{+}$ and $NO_{3}^{-}$ had positive associations at N agronomic practices both with and without wood biochar.

**Figure 4:**
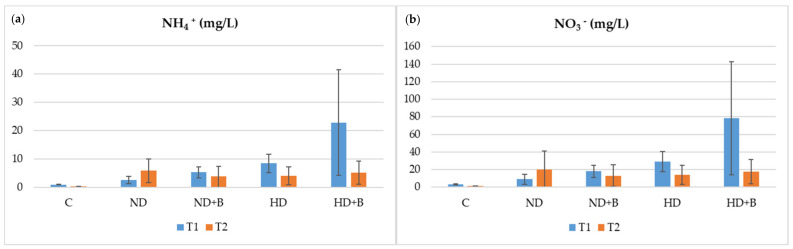
Results of ammonium N-leaching (**a**) and nitrate N-leaching (**b**) performance for different times and treatments. C—control; ND—normal dose; ND+B—normal dose with biochar; HD—high dose; HD+B—high dose with biochar. T1 indicates 10 days after the first treatment with nitrogenous fertilizers; T2 indicates 10 days after the second treatment. The bars represent the standard deviation.

The effect of biochar amendment on the concentration of the N compounds in the leachates was significant in biochar-amended soil with 130 kg N ha^−1^ at the end of cauliflower growing. As shown in Figure 4, the biochar application reduced the amount of the nitrogenous compounds in the water leached from the cauliflower mesocosms treated with different doses of N fertilizers. In particular, greater variations were observed in the mean values of the HD experimental conditions with and without biochar at 10 days after the first fertilization (Time 1) and at 10 days after the second fertilization (Time 2). The highest amount of ammonium leaching at the second fertilization was found in cauliflower mesocosms treated with 130 kg N ha^−1^ (ND). This was higher than all the other treatments (with or without biochar). Treating the soil with biochar decreased ammonium leaching, especially in the application with high rates of N fertilizer (Figure 4a). $NH_{4}^{+}$ leachate in HD+B was decreased from 22.83 mg/L (T1) to 5.16 mg/L (T2), and in the ND+B treatment reduced from 5.25 mg/L (T1) to 3.79 mg/L (T2). Moreover, significant differences in nitrate leachate were observed at the end of the treatments. Except for the ND mesocosm, the amount of $NO_{3}^{-}$ was decreased in all treatments. Nitrate leachate in biochar-amended soil reduced from 18.02 mg/L (T1) to 13.03 mg/L (T2) in ND+B, and 78.40 mg/L (T1) to 17.73 mg/L (T2) in HD+B. In soil treated with high rates of N fertilizer, nitrate amount declined from 29.05 mg/L (T1) to 14 mg/L (T2).

### 2.3. Effect of Biochar-Amended Soil with N Fertilizers on Vegetative Measurements

**Figure 5:**
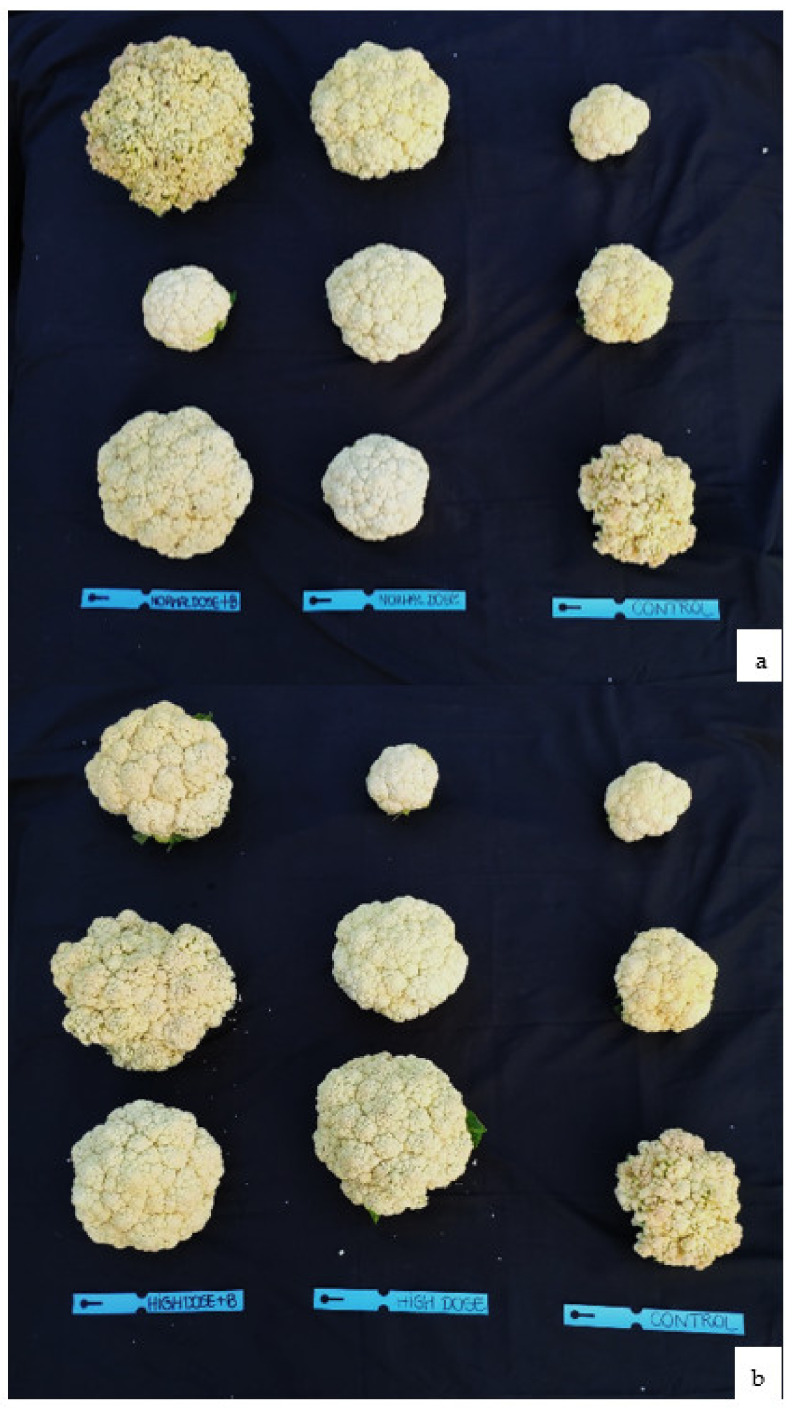
Cauliflower curd at the end of the vegetative growing season for the treatment with (**a**) a normal dose of N fertilizer (130 kg N ha^−1^) with and without biochar, and in comparison to the control, and (**b**) a high dose of N fertilizer (260 kg N ha^−1^) with and without biochar, and in comparison to the control. Each treatments type was conducted in triplicate.

**Figure 6:**
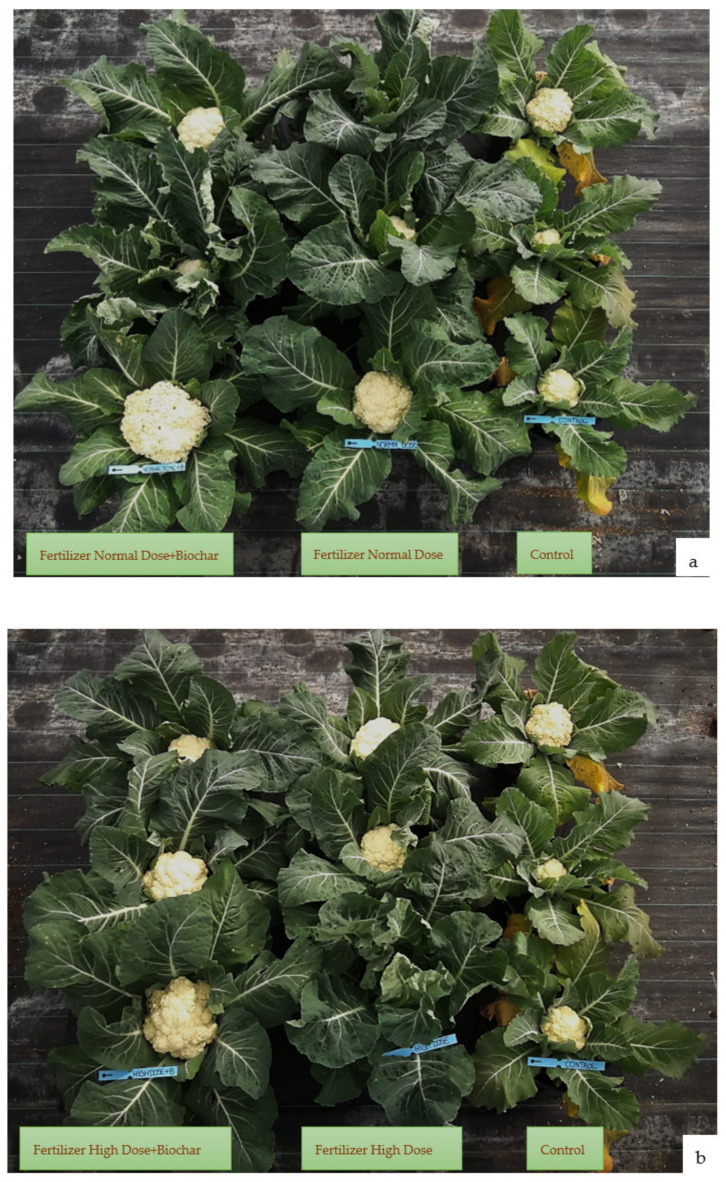
Cauliflower plants treated with 130 kg N ha^−1^ (**a**) and 260 kg N ha^−1^ (**b**) of fertilizer, with and without biochar, and compared to an untreated control.

The agronomic parameters assessed in the present study impacted plant morphological parameters at the end of the cauliflower growing season. As shown in Table 3, the biochar application did not affect measurements of cauliflower leaf tissue, or the Normalized Difference Vegetation Index (NDVI). More differences in curd size and weight were observed in biochar-amended soil (Figure 5). The plant vigor responded positively to fertilizer treatments of increasing nitrogen rates both with and without biochar (Figure 6). Compared with no biochar, the “green soil improver” increased the weight curd from 860.48 g (ND) to 880.68 g (ND+B), and from 729.14 g (HD) to 1097.60 g (HD+B). In the control samples (C), the average weight of curd was 370.37 g (Table 3). Additionally, the curd size was increased in the biochar-amended soils. In ND+B, the curd size was higher than ND with values equal to 19.50 cm and 18.71 cm, respectively. In the experiment with a high dose of N fertilizer, the biochar use improved the curd size from 6.17 cm (HD) to 21 cm (HD+B).

#### Principal Component Analysis and Cluster Analysis of Plant Growth Database

**Figure 7:**
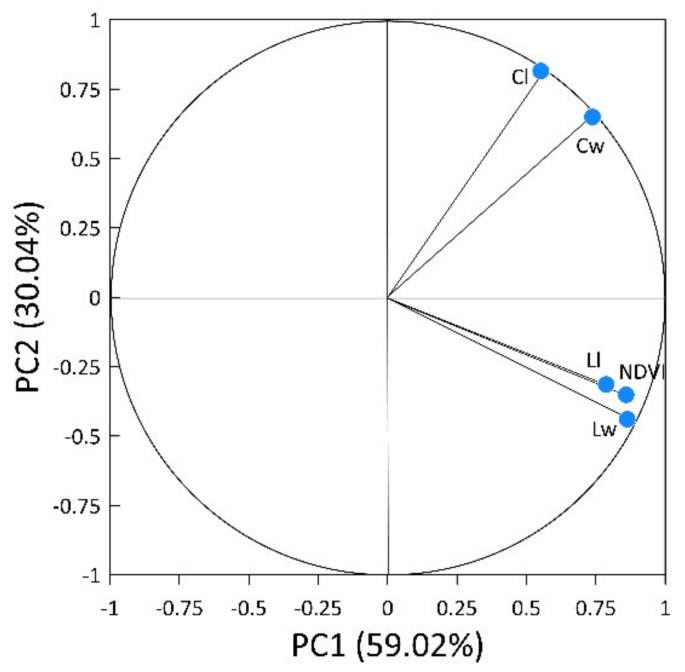
Principal component plot for the vegetative properties at the end of cauliflower growing.

For vegetative measurements in response to nitrogen fertilizing practices with or without biochar, two principal components (PC) were specified, explaining about 89% (PC1 59.02%; PC2 30.04%) of the cauliflower plant data total variance (Figure 7). The first component (PC1) was positively related to NDVI (0.787), curd weight (Cw, 0.738), leaf length (Ll, 0.859), and leaf width (Lw, 0.863). The second component, PC2, was positively related to curd size (Cs, 0.815).

**Figure 8:**
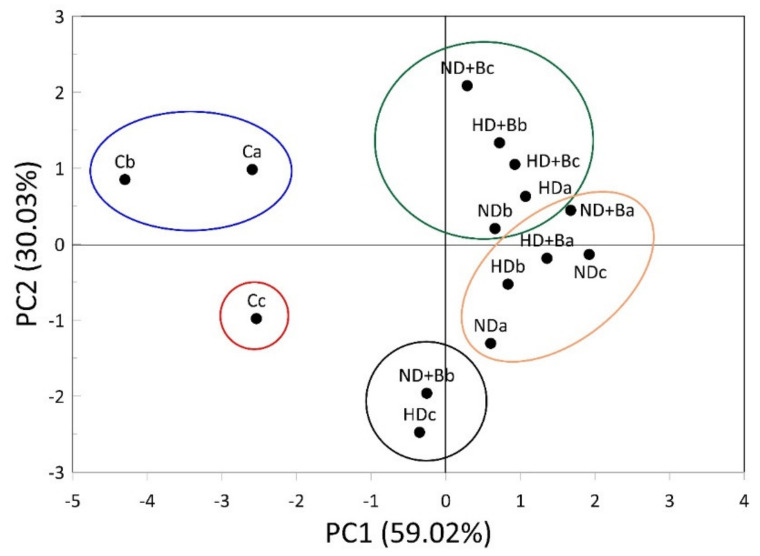
Hierarchical cluster analysis for the measurement of curd yield at the end of the growing season for different N fertilizer treatments with and without biochar. C—control; ND—normal dose; ND+B—normal dose with biochar; HD—high dose; HD+B—high dose with biochar; a-b-c indicate the triplicate samples.

Similar to that described in the soil property results, the application of the cluster analysis (CA) with PCA allowed the identification of five clusters for vegetative parameters (Figure 8).

The first cluster (C1) included two of the three replicate untreated controls (Ca and Cb), while the second cluster (C2) included the Cc replicate. The third cluster (C3) included two samples treated with a normal dose (ND) of fertilizer (Nda and Ndc), one sample treated with ND with biochar (ND+Ba), one sample treated with a high dose of fertilizer (HDb), and finally a sample treated with a high dose of fertilizer with biochar (HD+Ba). One sample treated with a normal fertilizer dose (NDb), one treated with a normal fertilizer dose with biochar (ND+Bc), one sample treated with a high fertilizer dose (HDa), and two treated with a double fertilizer dose with biochar application (HD+Bb, HD+Bc) represented the fourth cluster (C4).

Finally, the fifth cluster (C5) included a sample treated with a normal fertilizer dose with biochar (ND+Bb) and one treated with a high dose of N fertilizer (HDc).

No significance in the interaction of the variables was found (*p* = 0.2066 “ns”) for the vegetative measurements. ANOVA applied to each variable showed that only three variables (Table 3) had significant differences.

## 3. Discussion

Global nitrogen use increase is an environmental concern [37]. Sustainable agriculture can act as a solution to this problem, as it brings together different green technologies within a circular economy [38]. A potential strategy for nitrogenous compound mitigation in soil is its amendment with biochar. “Nitrate capture” relates to the uptake mechanisms of $NO_{3}^{-}$ by biochar, which is an object of ongoing scientific research. Most studies have reported the benefits of biochar amendment in soils, both in terms of improving plant performance and the environmental area, with a reduction in nutrient losses due to leaching [39,40,41]. The sustainability of cauliflower production may be improved by adopting biochar [42,43].

In the present study, wood biochar application in mineral nitrogen-fertilized soil was assessed as a green agricultural practice in order to study the effects of biochar on cauliflower yield, soil quality, and nitrogen compound quantification.

In our study, the use of biochar influenced the soil chemical properties of cauliflower mesocosms treated with different doses of N fertilizer (ND, HD, Table 1). The biochar application with 130 kg N ha^−1^ incremented the C/N ratio and $NO_{3}^{-}$ content. The addition of biochar in soil treated with 260 kg N ha^−1^ principally raised the TOC, total C, $NH_{4}^{+}$, and $NO_{3}^{-}$ values.

The effect of biochar application on soil pH was similar to that of nitrogen treatment in different doses (ND+B and HD+B). Different studies have demonstrated that soil pH increases due to biochar amendments in acidic soil [44,45], but its influence depends on the initial feedstock [46], soil type, and the type of crop grown. The level of pH in the ND+B was slightly higher than in the unamended soil (C), due to the liming effect of biochar [47,48]. Gonzaga et al. [49] also indicated small fluctuations in the pH of soil amended with pinewood biochar, highlighting the importance of biochar’s buffering capacity.

Soil electrical conductivity is a parameter influenced by feedstock biochar. At the end of the practice with N fertilizers, there was an increase in salinity in ND treatment compared to the biochar-untreated soil; the increase appeared to be of different magnitudes depending on the amount of N applied. The different saline responses of soils modified with biochar can be related both to the different levels of nutrient accumulation in the leaves of cauliflower plants, and the biochar’s adsorption capacity [50].

The response of total organic carbon (TOC) to N fertilizer treatment was highly significant. The highest level of TOC was observed in soil treated with 130 kg N ha^−1^ (30%, Table 1). In addition, in HD+B mesocosms, a higher mean value of TOC was observed than in the HD ones. The positive effects of biochar application leading to increased carbon content and carbon stocks, and improved biophysical–chemical properties of soil have been highlighted in numerous studies [44,51]. In our study, the value of TOC decreased only in treatment with ND and biochar. The reason for this could be that the TOC content decreases during the growing period of the crop [52], with a higher biochar decomposition rate [53]. As reported in Table 1, the effect of biochar application on soil C/N ratio was found to be significant in both treatments with different doses of N fertilizers.

Soil type, feedstocks, process conditions, amended nutrient type, processing fertilizer condition, and biochar dosage application are important elements that establish the adsorption and nutrient release pattern of crops [23]. Major changes in the total N were found in ND. The total nitrogen content fluctuations in biochar-treated mesocosms may have been associated with both a growing need for protein from soil bacteria, and plant growth [54,55]. Moreover, the lowest values of available P and total carbon contents were registered in ND+B. The difference in nutrient release rate by the biochar could have been due to the interaction of the base cations present in the soil with the nutrients and the pH of the soil [23].

Aside from variable surface chemistry, the biochar age is an important parameter that influences B sorption–desorption kinetics. Composted biochar addition had a significant effect on $NH_{4}^{+}$ content in HD+B. Several works found no direct correlation between biochar use and the concentration of ammonium in the soil [52], and biochar aging was found to coincide with an increase in more oxygenated functional groups, consequently affecting the kinetics of absorption [56].

Nitrate concentration in all mesocosms similarly decreased, showing no correlation with the application of biochar. These results could be associated with the high consumption of nitrates by cauliflower plants, and thus improved crop growth [50,52].

Some studies have indicated a considerable reduction in N leachate volume when biochar is used [57,58], associated with increased water retention, improved structure, and the aggregation of soil [59]. N-leaching is affected by soil type [60]. The leaching of ammonium is influenced by time and, in our study, the highest decreases were observed after the second fertilization (greater in ND+B), indicating a temporal trigger induced by biochar use [60]. The experiment induced a decrease in the amount of nitrate leachate in the water samples. However, significant increases in the N compounds of HD+B leachate with respect to the HD treatments were observed in the present study, possibly because the application of N at high rates could influence biochar’s capture capability. Borchard et al. [61] demonstrated that biochar utilization may induce an insignificant effect on the leaching yield of nitrate when the application rate of N exceeds 300 kg N ha ^−1^, due to the limited nitrate adsorption capacity of biochar. A maximum nitrogen fertilizer quantity of 260 kg N ha^−1^ was used in this work.

Soil aggregate structure is a significant factor in plant growth and the transport of water in soil [62]. This study found that the interaction of wood biochar and N inorganic fertilizer on the harvested cauliflower was significant (Table 3 and Figure 5). In all treatments with biochar, the size and weight of the curd were increased. This suggests that, compared with conventional fertilization alone, the application of 3% biochar could result in a high yield, as shown by several studies [63], with a view to sustainable agricultural practice.

Infrared-spectroscopy (FTIR) investigations are ongoing on cauliflower biomass samples (leaves and curds). This is in order to identify any potential effects of biochar on the amount and composition of chemical compounds in the crops, such as glucosinolates, which are important metabolites with potential anticancer properties.

Finally, next-generation sequencing analyses of the hypervariable V3–V4 regions of the 16S rRNA gene are in progress. This is to characterize the soil microbial community and, therefore, assess the effects of biochar addition on the soil biogeochemical cycle, and on the improvement of sustainable cauliflower crop production.

## 4. Materials and Methods

### 4.1. Experimental Conditions and Crop Production

**Figure 9:**
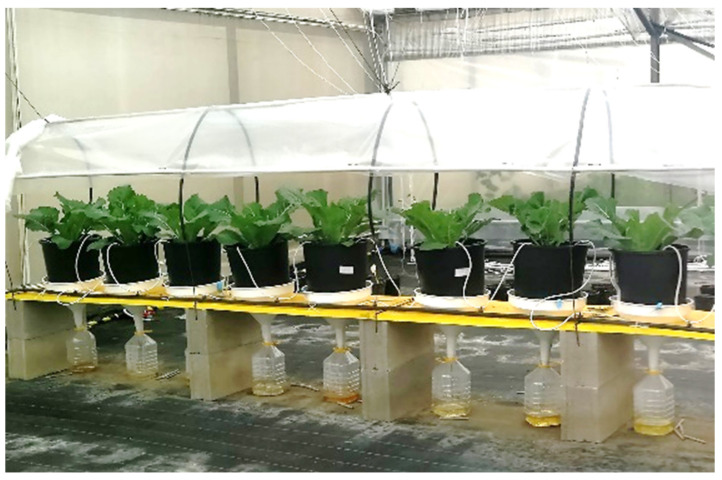
Preparation of the cauliflower plant growth experiment with water leaching collection bottles.

The agronomic experiment was conducted at ReAgri S.r.l. (Massafra, Taranto, Italy) from October to February 2021. *Brassica oleracea* L. var. *botrytis*. (Akara, Syngenta) plants were grown in a greenhouse over a natural photoperiod, with a plastic cloth to avoid the addition of rainwater. As shown in Figure 9, seedlings were transplanted in pots equipped with plastic bottles for the collection of leached water.

**Figure 10:**
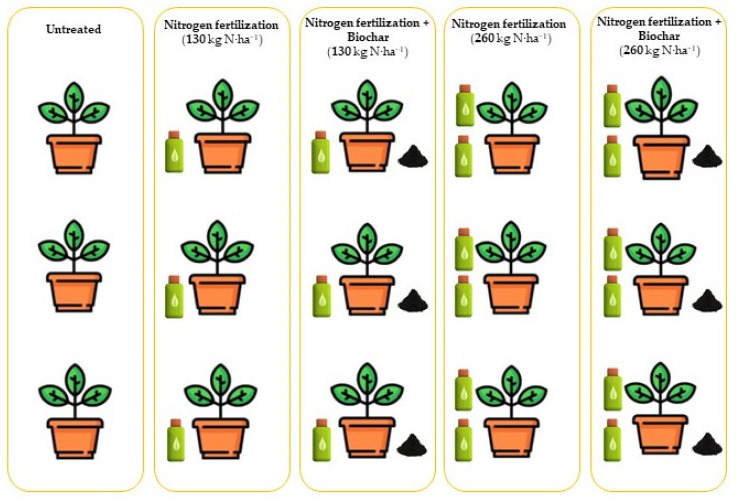
Experimental design showing the treatment types.

Mineral nitrogen fertilizer application was carried out at (1) a conventional application rate (130 kg N·ha^−1^), defined as normal dose (ND), and (2) a high application rate (260 kg N·ha^−1^), defined as high dose (HD), in comparison with biochar-amended soils. The control was untreated (Figure 10). Mineral N fertilizer (calcium nitrate 14.4% N) was applied to the 0–30 cm soil layer. In biochar-amended soils, we added mixed woody waste biochar (800–900 °C, Syngasmart, Rieti, Italy to the soil, in a ratio of 3% compared to the total volume. The physical and chemical properties of the biochar are given in Table 4.

Nitrogen fertilization was divided into two phases. Specifically, the agricultural practice was performed 8 weeks after transplantation (flowering induction requires 3–6 kg N /ha/day) and 20 weeks after transplantation (inflorescence enlargement requires 3–4 kg N/ha/day).

At the beginning of the experiment, biochar was amended with the feedstock soil (soil depth of about 15 cm). Nitrogen fertilization was carried out manually.

The test was finished when the curds of the Brassicaceae reached the size required by the market.

### 4.2. Sample Collection

Soil samples from all mesocosms were collected from between 0 and 20 cm at the end of the growing cauliflower season. Soil samples were conserved at 4 °C and processed within 1 month. Water samples were collected about 10 days after treatment from each plastic bottle (for a total number of 15) and were filtered through 0.45 µm pore size membrane filters for chemical analysis. In this case, sampling was timed to coincide with two critical phenological and agronomic periods in which cauliflower nitrogen demand is high, and in which growers generally add extra nutrients. The leaves and curds were harvested at the end of the experiment for agronomic parameter determination (weight curd, size curd, leaf length, and leaf width).

### 4.3. Soil Chemical Properties

The soil chemical properties were investigated before the mesocosms were set up and after each fertilization. The determinations of pH value, water content percentage, and electrical conductivity were performed according to the Italian Official Methods of Soil Chemistry approved by the Minister for Agricultural Policies [64]. Available phosphorus (AvP) was determined spectrophotometrically starting from an aqueous soil extract according to the Olsen Method [65], while total organic carbon (TOC) was analyzed by a TOC analyzer (TOC-L, Shimadzu, Kyoto, Japan). The percentage of carbon and nitrogen in soil and the C: N ratio were also measured using an elemental analyzer (Thermo Flash 2000, CHNS-O Analyzer, Thermo Scientific, Eindhoven, The Netherland).

### 4.4. Nitrate and Ammonium Quantification

Inorganic N was extracted from the soil with 2 M KCl [66] on a shaker for 1 h at room temperature (20 °C). Tubes were centrifuged (4500× *g*, 10 min) and the supernatant was decanted into clean cylinders. Aliquots were taken for the quantification of nitrate and ammonium. Nitrate was quantified by ionic chromatography with the Metrohm 930 compact IC flex [67], and ammonium by spectrophotometry of the soil and water samples with the PerkinElmer spectrometer Lambda 950 [68].

### 4.5. Plant Analyses

At harvest, all plants were sampled and a range of yield parameters were measured (weight curd, size curd, leaf length, and leaf width). Moreover, the Normalized Difference Vegetation Index (NDVI) was estimated at a distance of 1 m from the plants with a Trimble Green-Seeker handheld crop sensor.

### 4.6. Statistical Analyses

To create a dataset of the soil samples and vegetative properties, principal component analysis (PCA) and cluster analysis were carried out. The application of the PCA involved the simultaneous analysis of several variables concerning their reciprocal relations. The main purpose of a PCA is to reduce a large dataset with many variables to a simplified dataset of a few major components that describe most of the original variance.

For the soil matrix, the variables used were pH, EC, water content percentage, P available, TOC, C/N, N total, C total, $NH_{4}^{+}$, and $NO_{3}^{-}$. A hierarchical cluster analysis was applied to the scores of the principal component analysis (PCA), allowing the classification of the agronomic treatments into different groups.

PCA and cluster analysis were also applied to the cauliflower vegetative measurements dataset, of which the variables were NDVI, card weight and length, and leaf length.

To determine the variability of the investigated soil properties and plant characteristics in response to the different soil treatments (nitrogen fertilizing practices with or without biochar), multivariate analysis of variance (MANOVA) was performed using Pillai’s trace test to test the statistical differences. This test is considered to be a powerful and robust statistical measure. When the MANOVA test was significant, the univariate analysis of variance (ANOVA) was performed on each variable and factor to determine which of them influenced the significance.

With the aim of identifying the ability of wood biochar to retain nitrogen compounds in soils treated with nitrogen fertilizers, simple linear regression was performed on the water samples after two fertilizing practices.

## 5. Conclusions

Biochar use in the cauliflower mesocosms assessed in this study improved the curd yield of cauliflower cultivated in South Italy in conjunction with the conventional rates of nitrogen fertilizer. Biochar enhanced nutrient availability in the soil by improving its chemical and physical properties. Biochar reduced leached nitrogen, and thus has potential to mitigate the pollutive effects that stem from the mass use of nitrogen fertilizers. The highest values of curd size and weight were obtained at the biochar rate of 3%. Moreover, biochar-amended soil treated with a conventional N fertilizer dose increased the N compounds in the soil and decreased them in the leached water samples.

## Figures and Tables

**Table 1:** Effect of biochar on soil chemical properties at the end of the cauliflower growing season. C—control; ND—normal dose; ND+B—normal dose with biochar; HD—high dose; HD+B—high dose with biochar; EC—electrical conductivity; TOC—total organic carbon; s.d.—standard deviation; a-b-c indicate the triplicate samples. The last row includes ANOVA results: * = the difference is significant at *p* < 0.05; ** = the difference is significant at *p* < 0.01; *** = the difference is significant at *p* < 0.001.

Sample	pH	EC	Water Content	Available P	TOC	C/N	Total N	Total C	$NH_{4}^{+}$	$NO_{3}^{-}$
		(dS/m^−1^)	(%)	(mg kg^−1^)	(%)		(%)	(%)	(mg/L)	(mg/L)
C_a	4.98	1.60	16.28	22.86	10	99	0.5	32.3	1.50	0.00
C_b	5.38	1.58	23.35	28.57	8	114	0.2	24.8	1.61	0.00
C_c	5.07	1.55	17.5	28.57	9	135	0.2	31.2	1.15	0.00
mean	5.14	1.58	19.04	26.67	8.94	116.09	0.29	29.42	1.42	0.00
s.d.	0.21	0.03	3.78	3.30	0.91	18.33	0.16	4.05	0.24	0.00
ND_a	5.21	2.38	14.42	28.57	30	124	0.4	33.4	5.63	9.88
ND_b	5.23	2.27	15.72	28.57	19	118	0.4	33.5	3.18	24.40
ND_c	5.27	2.30	11.96	28.57	17	132	0.4	38.1	12.33	31.75
mean	5.24	2.32	14.03	28.57	21.89	124.71	0.4	34.99	7.05	22.01
s.d.	0.03	0.06	1.91	0.00	7.00	7.09	0.03	2.68	4.74	11.13
ND+B_a	5.22	2.72	8.94	17.14	19	119	0.2	21.7	3.17	64.69
ND+B_b	5.4	2.62	13.59	17.14	3	133	0.2	28.0	5.45	37.71
ND+B_c	5.1	2.65	11.34	17.14	22	140	0.2	26.8	5.24	43.87
mean	5.24	2.66	11.29	17.14	14.83	130.61	0.2	25.5	4.62	48.76
s.d.	0.15	0.05	2.33	0.00	10.02	10.62	0.03	3.35	1.26	14.14
HD_a	4.78	4.35	10.46	20.00	11	113	0.2	25.7	29.98	73.41
HD_b	5.2	4.35	13.42	20.00	11	89	0.3	22.4	/	82.52
HD_c	5.12	4.67	11.47	20.00	13	116	0.3	30.8	6.46	143.13
mean	5.03	4.46	11.78	20	11.67	106.08	0.3	26.31	18.22	99.69
s.d.	0.22	0.18	1.50	0.00	0.98	15.13	0.06	4.23	16.63	37.90
HD+B_a	5.01	3.52	11.73	20.00	24	116	0.1	21.8	28.62	61.92
HD+B_b	4.98	3.61	10.41	20.00	15	119	0.4	41.6	24.69	77.17
HD+B_c	4.92	3.59	12.39	20.00	11	115	0.3	28.8	24.69	93.79
mean	4.97	3.57	11.51	20	16.74	116.6	0.27	30.75	26	77.63
s.d.	0.05	0.05	1.01	0.00	6.44	2.12	0.16	10.04	2.27	15.94
*p* -value	0.1992	3.46 × 10^−11^ ***	0.00981 **	9.954 × 10^−11^ ***	0.1852	0.2084	0.31	0.2982	0.0178 *	0.00075 ***

**Table 2:** Factor loadings of the first two main components of the PCA for variable soil parameters associated with nitrogen fertilizer application with and without biochar.

Soil Chemical Properties	Factor 1	Factor 2
pH	−0.614	−0.317
EC	0.902	0.052
Water content percentage	−0.799	−0.244
Available P	−0.775	0.333
TOC	0.069	0.308
C/N ratio	−0.242	0.036
Total N	−0.216	0.817
Total C	−0.268	0.894
$NH_{4}^{+}$	0.673	0.287
$NO_{3}^{-}$	0.869	0.038

**Table 3:** Effect of biochar application on vegetative measurements at the end of the cauliflower growing season. The last row includes ANOVA results: * = the difference is significant at *p* < 0.05; ** = the difference is significant at *p* < 0.01.

Sample	NDVI	Cw	Cs	Ll	Lw
		(g)	(cm)	(cm)	(cm)
C_a	0.63	518.7	18	26	13.5
C_b	0.55	345.96	14	26.5	4.5
C_c	0.74	246.16	11.5	26.5	15
mean	0.64	370.27	14.50	26.33	11.00
s.d.	0.10	137.89	3.28	0.29	5.68
ND_a	0.76	639.72	15	54	28
ND_b	0.78	1026.19	18	45.5	22.5
ND_c	0.79	915.52	21.5	59	28.5
mean	0.78	860.48	18.17	52.83	26.33
s.d.	0.02	199.03	3.25	6.83	3.33
ND+B_a	0.73	1111.57	21.5	56.5	29
ND+B_b	0.7	405.53	12	55	28.5
ND+B_c	0.74	1124.94	25	36.5	18
mean	0.72	880.68	19.50	49.33	25.17
s.d.	0.02	411.55	6.73	11.14	6.21
HD_a	0.8	1138.59	20	45	22.5
HD_b	0.79	761.72	18	51	25
HD_c	0.78	287.12	10.5	46	29
mean	0.79	729.14	16.17	47.33	25.50
s.d.	0.01	426.67	5.01	3.21	3.28
HD+B_a	0.78	969.5	19	51	28.5
HD+B_b	0.69	1115.42	23	45	25.5
HD+B_c	0.74	1207.88	21	45	24
mean	0.74	1097.60	21.00	47.00	26.00
s.d.	0.05	120.18	2.00	3.46	2.29
*p* -value	0.0264 *	0.1056	0.4291	0.00296 **	0.00689 **

C—control; ND—normal dose; ND+B—normal dose with biochar; HD—high dose; HD+B—high dose with biochar; NDVI—Normalized Difference Vegetation Index; Cw—curd weight; Cs—curd size; Ll—leaf length; Lw—leaf width; s.d.— standard deviation; a-b-c indicate the triplicate samples.

**Table 4:** Chemical and physical properties of the biochar used in the study.

Parameter	Unit	Value
Total nitrogen content	%	0.5
Potassium content	%	0.4
Phosphorous content	%	0.3
Calcium content	%	1.1
Magnesium content	%	0.2
Sodium content	%	0.2
Organic carbon content	mg/kg	68.40
pH (H_2_O)	-	11.3
Electrical conductivity	dS/m	5.0

## Data Availability

The data presented in this study are available on request from the corresponding author.
